# Regulation of Eosinophilia in Asthma—New Therapeutic Approaches for Asthma Treatment

**DOI:** 10.3390/cells10040817

**Published:** 2021-04-06

**Authors:** Ruth P. Cusack, Christiane E. Whetstone, Yanqing Xie, Maral Ranjbar, Gail M. Gauvreau

**Affiliations:** Department of Medicine, McMaster University, Hamilton, ON L8S 4K1, Canada; cusackr@mcmaster.ca (R.P.C.); whetstoc@mcmaster.ca (C.E.W.); xiey56@mcmaster.ca (Y.X.); ranjbm1@mcmaster.ca (M.R.)

**Keywords:** eosinophil, asthma, clinical trials, therapy, inflammatory mechanisms

## Abstract

Asthma is a complex and chronic inflammatory disease of the airways, characterized by variable and recurring symptoms, reversible airflow obstruction, bronchospasm, and airway eosinophilia. As the pathophysiology of asthma is becoming clearer, the identification of new valuable drug targets is emerging. IL-5 is one of these such targets because it is the major cytokine supporting eosinophilia and is responsible for terminal differentiation of human eosinophils, regulating eosinophil proliferation, differentiation, maturation, migration, and prevention of cellular apoptosis. Blockade of the IL-5 pathway has been shown to be efficacious for the treatment of eosinophilic asthma. However, several other inflammatory pathways have been shown to support eosinophilia, including IL-13, the alarmin cytokines TSLP and IL-33, and the IL-3/5/GM-CSF axis. These and other alternate pathways leading to airway eosinophilia will be described, and the efficacy of therapeutics that have been developed to block these pathways will be evaluated.

## 1. Introduction

Asthma is an inflammatory condition of the airways that results in recurring symptoms such as airway obstructions, bronchial spasms, and elevated levels of eosinophils in the airways (eosinophilia). One of the hallmarks of asthma is the presence of eosinophils, which accumulate in the airways where they are believed to be an essential effector cell in the pathogenesis of the allergic disease. Migration and proliferation of eosinophils depend on the highly regulated signaling of several chemokines and cytokines. Elevated levels of the cytokines IL-3, IL-5, and GM-CSF, together with thymic stromal lymphopoietin (TSLP), IL-4, IL-13, IL-17, IL-25, IL-33, and chemokines acting through the chemokine receptor CCR3, cause extensive inflammation, mucosal remodeling, and mucus hypersecretion in the airways [1]. Despite eosinophils persisting at relatively low levels in the bloodstream, accounting for approximately 5% of the total white blood cell count in asthma [2], eosinophil counts increase drastically in the asthmatic airways after bronchoprovocation [3], and are found at high levels in airways of most patients with chronic asthma [4]. IL-5 is the major cytokine supporting eosinophilia, responsible for terminal differentiation of human eosinophils, and regulates eosinophil proliferation, differentiation, maturation, migration, and prevention of cellular apoptosis. Anti-IL-5 therapy as a method of asthma treatment has been introduced over the past decade for the treatment of severe eosinophilic asthma. However, several other inflammatory pathways have been shown to support eosinophilia, including IL-13, and most recently, the alarmin cytokines TSLP and IL-33. This review will describe the mechanisms and efficacy of various therapeutics and biologics targeting eosinophilic airway inflammatory pathways, including glucocorticosteroids, therapeutics targeting specific receptors on eosinophils, T2 cytokines, and upstream pathways. Table 1 summarizes investigational therapies that have been or are currently undergoing evaluation for treatment of asthma.

## 2. Glucocorticosteroids

Glucocorticosteroids (GCs) are the most common and effective anti-inflammatory drugs used for the treatment of airway diseases, including asthma. The actions of glucocorticoids are mediated through glucocorticoid receptors (GR) that are expressed throughout the body. Upon activation of GR receptors through direct binding to DNA response elements and/or physical association with other transcription factors, the transcription of genes is either induced or repressed. Heterogeneity in glucocorticoid sensitivity and biological responses exists across tissues, mainly due to a diverse collection of receptor isoforms [5]. As such, the mechanism of action of GR agonists in asthma is nonspecific and impacts a wide variety of cells within the airways to suppress airway inflammation, prevent the recruitment of inflammatory cells to the airways, and indirectly promote the relaxation of smooth muscles [6,7,8].

Essential actions of inhaled glucocorticosteroids (ICSs) for the treatment of eosinophilic asthma include the prevention of eosinophil recruitment from the bone marrow and migration into the airways, as well as suppressing the expression of eosinophil survival factors and inducing eosinophil apoptosis [9]. GCs suppress eosinophil maturation by tempering the production and/or release of IL-3, IL-5, GM-CSF, and other eosinophil factors from cells within the bone marrow. Eosinophil proliferation and maturation are indirectly blocked through the suppression of cytokine release from accessory cells [10]. Glucocorticoids are reported to block the upregulation of specific adhesion molecules such as ICAM-1 and CD18 on eosinophils [11,12]. Certain cytokines, especially IL3, IL-5, GM-CSF, and IFN-γ, prolong eosinophils’ survival; however, when exposed to glucocorticoids, eosinophils undergo apoptosis, even in the presence of these cytokines [13,14,15]. Aside from eosinophils, GCs are proven to have broader impacts on other components of the immune system, leading to reduced manifestation in asthmatic patients. GCs inhibit lymphocyte activation and inflammatory mediator expression and induce lymphocyte apoptosis, actively reducing total blood lymphocyte numbers in asthmatics [16]. GCs have profound effects on the functionality, terminal differentiation, and activation status of macrophages and monocytes in asthma, reducing the expression of macrophage-derived proinflammatory cytokines and chemokines [17]. ICS treatment also reduces peripheral blood levels of monocytes and low-affinity IgE receptor expression [18]. Dendritic cells (DC) can be regulated by ICS through CCR7 expression, hampering DC migration to local lymphoid collections [19]. Finally, GCs suppress the release of Th1 and Th2 polarizing cytokines [20,21].

In contrast to the suppression of most innate immune-inflammatory responses in the airways, GCs seem to have little effect on neutrophil production and survival [22], in addition to macrophage phagocytosis and epithelial cell survival, and have been shown to increase the expression of Toll-like receptors, complement, pentraxins, collectins, SAA, and other host defense genes [23,24]. Historically, routine use of ICS to prevent airway inflammation in combination with rescue relievers such as β_2_agonists to relax smooth-muscle contractions are the most successful treatment for controlling asthma symptoms, reducing exacerbation, increasing lung function, and overall improving asthma control and quality of life. However, a small subset of asthmatic patients responds poorly to GC treatment, which is associated with neutrophilic airway inflammation [25]. While eosinophilic inflammation is known to be associated with better disease outcomes with ICS therapy, recent studies have shown that severe asthmatics with late-onset eosinophilic phenotype tend to have persistent airway inflammation even after GC therapies [26,27,28,29,30]. New and more specific GR agonists are being developed for more specific regulation of genes known to drive asthma [31].

## 3. Targeting T2 Cytokines

### 3.1. The IL-3/5/GM-CSF Axis

Growth factors IL-3, IL-5, and GM-CSF together are essential for inducing eosinophil differentiation from CD34+ pluripotent hematopoietic stem cells (HSCs), which are bone marrow progenitor cells [32]. These pleiotropic regulators are also critical for eosinophil survival, migration, and activation [33,34,35], to maintain steady-state hematopoiesis and regulation of inflammation in response to triggers such as pathogens, autoimmune disease, and cancer. From the HSCs, common myeloid progenitors differentiate into granulocytes/macrophage progenitors (GMPs), megakaryocyte/erythrocyte progenitors (MEPs) or CD34 + IL-5Rα + eosinophil lineage-committed progenitors (EoPs) [36]. IL-3 and GM-CSF are crucial in the early-stage differentiation of eosinophil progenitors from CD34+ cells, while IL-5 is required for their final maturation [37,38]. Eosinophils in immature states may concurrently develop locally within tissues or sites of infections via in situ hematopoiesis [39]. Lineage-committed eosinophil precursors, CD34 + /IL-5Rα + precursor cells, are found at significantly higher concentrations in peripheral blood and bronchial biopsies of atopic asthmatics as compared to atopic and nonatopic nonasthmatic controls [37,40]. Eosinophil progenitors from the blood of patients with severe eosinophilic asthma have exaggerated clonogenic responses to IL-5 in vitro [34].

Despite eosinophils persisting at relatively low levels in the bloodstream, accounting for approximately 5% of the total white blood cell count in asthma [2], eosinophil counts increase drastically in the asthmatic airways after bronchoprovocation [3], and are found at high levels in airways of most patients with chronic asthma [4]. IL-3, IL-5, and GM-CSF contribute to eosinophil survival in tissue by delaying eosinophil apoptosis [41,42,43], as well as inducing eosinophil activation through degranulation [44,45,46,47]. Once eosinophils infiltrate a site of inflammation, stored mediators including toxic granule proteins, cytokines, chemokines, and growth factors are released during eosinophil degranulation [48].

**Figure 1:**
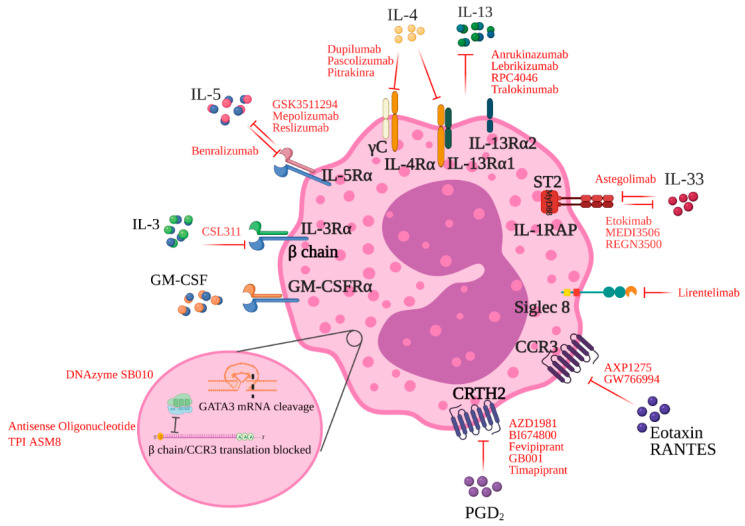
Eosinophil-targeted therapies and their ligands.

IL-3, IL-5, and GM-CSF have heterodimeric receptors composed of a common β receptor chain (β c) and an individual α chain. These cytokines bind with low affinity (nanomolar) to their respective α-chain, and recruitment of the β -chain results in a conformation change to a high-affinity (picomolar) binding complex [49]. The IL-5Rα expression is typically limited to eosinophils and basophils, making it an ideal therapeutic target to reduce eosinophilia in allergic diseases (Figure 1).

The first class of eosinophil-targeted biological treatments, mepolizumab and reslizumab anti-IL-5 antibodies, aimed to reduce the circulating IL-5 cytokine levels and inhibit its receptor association through configural changes to the cytokine, respectively. Mepolizumab is a humanized monoclonal *N*-glycosylated IgG1/k antibody that binds to the α-chain of circulating IL-5, preventing its association with the α subunit of the IL-5 receptors. Reslizumab is an IgG4/k humanized monoclonal antibody blocking circulating IL-5, preventing it from binding to eosinophil receptors [50]. In phase 3 clinical trials, mepolizumab (DREAM, MENSA, and SIRIUS) [51,52,53] and reslizumab (2 BREATH studies) [54] showed clinically significant reductions in exacerbations by approximately half in patients with severe eosinophilic asthma on the standard of care (at least medium-dose inhaled corticosteroids) with a poorly controlled disease (either two or more exacerbations in the preceding year of Asthma Control Questionnaire 1.5 or more). Both anti-IL-5 treatments produced small yet statistically significant improvements in mean prebronchodilator forced expiratory flow in one second (FEV_1_). Mepolizumab and reslizumab were shown to have statistically significant effects at lower sputum eosinophil levels, yet had relatively smaller success at reducing blood eosinophil levels.

Benralizumab (IgG1/k) takes a unique approach by directly binding the α subunit of the IL-5 receptor-bearing cells such as eosinophils and basophils, thereby hindering eosinophilopoiesis, as well as eosinophil maturation and survival [50,55]. In addition, having a high affinity to human FcyRIIIα, benralizumab induces apoptosis through antibody-dependent cell-mediated cytotoxicity (ADCC), whereby natural killer cells target and deplete IL-5 receptor alpha-bearing cells [46]. This approach avoids autoimmune-mediated worsening of asthma, which has previously been reported with low dose anti-IL-5 therapy [56,57]. Phase 3 clinical studies (SIROCCO, CALIMA, ZONDA, BISE) verified the use of benralizumab for severe, uncontrollable, high eosinophil blood count patients. Exacerbation rates were significantly reduced by as much as 70% (ZONDA). Statistically significant increases in FEV_1_ were observed, as well as a 75% reduction in the oral glucocorticoid dose required by patients after treatment ceased (ZONDA) [58,59,60,61]. Blood eosinophil counts were reduced to below detection levels throughout treatment, and patients with blood eosinophil counts of >300 cells/mL responded better than patients with blood eosinophil counts of <300 cells/mL. Although studies have suggested benralizumab as a promising biologic for asthma exacerbations, the clinical efficacy of benralizumab as compared to other IL-5 targeted therapies has not been established. However, Busse et al. and colleagues have reported that mepolizumab might be the better option compared to other anti-IL-5 antibodies. By indirect treatment comparison method and comparing the baseline eosinophil counts between patients receiving benralizumab, mepolizumab, and reslizumab, mepolizumab significantly improves the exacerbations in asthmatic patients [62]. Contrary to what was reported by Busse et al., Bourdin et al. reported similar efficacy for benralizumab and mepolizumab [63]. These conflicting results can only be addressed by direct comparisons of various therapies.

Although recent studies of anti-IL-5 therapies have led to a reduction in exacerbations and improvement of several asthma-control measures in a subpopulation of patients displaying severe eosinophilic asthma, there are other approaches for regulating eosinophils. Some therapies have been developed to target the common βc (βc, βcR, CD131, CSF2RB), which could effectively inhibit the activity of all three βc-signaling cytokines and affect the functionality of the hematopoietic cells they regulate. Studies of βc- and IL-3-specific β homodimer (β_IL-3_)-deficient mouse models of allergic inflammation (βc^−/−^ and β_IL-3_^−/−^ designated βc^−/−^ mice) demonstrated that signaling through βc by IL-3, IL-5, and GM-CSF is critical for the development and functionality of immune cells in the inflammatory response [64].

CSL311 is a novel IgG4/k human monoclonal antibody that binds to a unique epitope specific to the cytokine-binding site of the human β common (βc, βcR, CD131, CSF2RB), aiming to specifically and effectively target inflammation mediated by IL-3, IL-5, and GM-CSF. CSL311 has a picomolar binding affinity for the human βc receptor and, at therapeutic concentrations, is a highly potent antagonist of the united activities of IL-3, IL-5, and GM-CSF on eosinophil survival. CSL311 treatment in vitro has been shown to inhibit the survival of sputum-derived inflammatory cells collected from asthmatic patients undergoing allergen bronchoprovocation [65]. More recently, using a humanized mouse xenograft model, CSL311 was shown to inhibit human nasal polyp pathophysiology [66], further supporting a therapeutic role in eosinophilic disease. Currently, CSL311 is being evaluated for safety and tolerability in a phase 1 study conducted in patients with mild asthma (NCT04082754).

### 3.2. IL-4 and IL-13 Blockade

Other type 2 cytokines such as IL-4 and IL-13 are thought to contribute to eosinophilic airway disease through regulating the responses of lymphocytes, myeloid cells, and nonhematopoietic cells. Relating to asthma, IL-4 is known to induce the differentiation of naïve CD4 T cells into Th2 cells and drives the immunoglobulin (Ig) class switch to IgG1 and IgE in B cells [67]. IL-13 appears to have broader immunoregulatory and effector roles in allergic diseases such as bronchial asthma [68,69]. A variety of immune and nonimmune cells are known as IL-13 producers, including T cells, mast cells, basophils, dendritic cells, and keratinocytes [70,71,72]. IL-13 has been suggested to be a chemotactic factor, an activator, and a survival factor for eosinophils [73,74]. IL-13 promotes eosinophilic inflammation in part by upregulating the expression of eosinophil-attracting CCR3-binding chemokines. IL-13 also promotes leucocytes and resident airway cells to induce the CCR4-binding chemokines, which are increased in allergic asthma patients [75,76]. While eosinophils do not constitutively express IL-13, they have been shown to inducibly synthesize this cytokine upon stimulation with cytokines IL-5 and GM-CSF. As such, eosinophils in the T2 microenvironment, such as the asthmatic airway, could contribute to IL-13 production [77].

The cytokine-binding receptor chain for IL-4 is IL-4Rα, which is widely expressed. The IL-4/IL-4Rα-complex binds a secondary receptor chain, either IL-2Rγc (γc) or IL-13Rα1. In contrast, the IL-13 receptor has two separate binding chains; namely, IL-13Rα1 and IL-13Rα2. Formation of a type I or a type II IL-4 receptor is determined after the IL-4/IL-4Rα complex is formed, whereas binding of IL-13 upon either IL-13Rα1 or IL-13Rα2 determines which receptor IL-13 utilizes. Anti-IL-13 therapies have been developed to block the IL-13 cytokine to interfere with binding to IL-13Rα1 (IMA-026, tralokinumab), to interfere with binding to IL-4Rα (IMA-638, lebrikizumab), or to block the IL-4Rα where it binds to IL-4 and IL-13 (dupilumab), thus inhibiting IL-4 and IL-13 signaling (Figure 1). Allergen challenge studies in humans with mild allergic asthma have shown that interfering with IL-4Rα binding [78,79,80], but not IL-13Rα1 binding [78], attenuates allergen-induced late asthmatic response. In contrast, none of these anti-IL-13 monoclonal antibodies had any effect on eosinophil levels in blood or airways. A mechanistic study of lebrikizumab (IgG4) in moderate-to-severe uncontrolled asthma demonstrated no change in eosinophil numbers in the bronchial mucosa. However, lebrikizumab reduced subepithelial fibrosis, a feature of airway remodeling [81]. Tralukinumab (IgG4) and lebrikizumab asthma programs were both discontinued after failing in phase 2 and 3 trials, respectively [82,83,84]. Dupilumab (IgG4) met primary endpoints (occurrence of an asthma exacerbation, change in FEV1 in patients with baseline blood eosinophil counts of at least 300 eosinophils per μL) for phase 3 studies in adults and children 12 years and older [85,86,87,88], showing elevated blood eosinophil levels after injection, which is presumably justified by the suppression of chemokine generation, leading to decreased recruitment of blood eosinophils into the lung tissue [89]. 

## 4. Targeting Specific Receptors on Eosinophils

### 4.1. CCR3 Blockade

Other approaches for blocking eosinophil migration have targeted receptors of potent eosinophil chemokines. CCR3 is the cognate receptor for major human eosinophil chemoattractants, expressed by eosinophils [90] and important for their recruitment to the lung through its binding to eotaxin [91,92,93]. A number of other chemokines from the eotaxin family of proteins, including RANTES; MIP-1; and MCP-2, 3, and 4; many of which are elevated in asthma and correlate with disease severity, also bind to CCR3 (Figure 1). Although the eotaxin–CCR3 pathway is required for eosinophil trafficking, the CCR3 receptor is also present on other cells known to have effector functions in asthma, including basophils, mast cells, CD34 + cells, airway epithelial cells, and activated T cells [94]. With CCR3 having an association with many of the cells and chemokines involved in asthma and allergy, it is theorized that blockade of this receptor may have marked effects in eosinophilic diseases.

AXP1275 is a CCR3 receptor antagonist that has been evaluated for efficacy in the human allergen challenge model of asthma. This disease model had been widely used to examine efficacy of asthma drugs because (1) it mimics environmental allergen exposure; (2) it drives a cascade of inflammatory events, including IgE- and T2-mediated cellular inflammation; (3) it enhances well-defined features of asthma such as airway inflammation, bronchoconstriction and airway hyperresponsiveness that are responsive to anti-inflammatory therapy; and (4) experiments are conducted in steroid-naïve allergic mild asthmatics, which allows for investigation therapy to be assessed without confounding effects of background standard asthma therapy [3,95,96,97]. A two-week treatment regime of oral CCR3 antagonist followed by an inhaled allergen challenge in mild allergic asthmatics resulted in a trend, but no statistically significant reduction in allergen-induced airway eosinophils, with no change in physiological outcomes of allergen-induced bronchoconstriction [98]. It is possible that AXP1275 was not present at high enough concentrations to prevent allergen-induced cell migration, or that chemokines signaling through other receptors such as CCR2 and CCR5 helped to drive eosinophil migration postallergen challenge. There was, however, a significant improvement in airway hyperresponsiveness to methacholine. Interestingly these results were substantiated by another clinical trial using a different oral CCR3 antagonist in patients with asthma and eosinophilic bronchitis, showing that despite 90% receptor occupancy, there was no improvement in eosinophil counts in blood or airways, yet a modest and statistically significant improvement in methacholine PC20 [99]. These studies raise questions about the role of CCR3 in airway eosinophilia, and suggest CCR3-mediated mechanisms could be a factor in airway hyperresponsiveness of patients with asthma.

### 4.2. CCR3 and Common β-Chain Blockade

With considerable redundancy in receptors and chemokines involved in eosinophil recruitment, control of eosinophil levels may be more effective using a multifaceted approach that simultaneously blocks more than one mechanistic pathway. TPI ASM8 was developed as a drug composed of two modified phosphorothioate antisense oligonucleotides: TOP004, directed against the human common βc of IL-3, IL-5, and GM-CSF receptors; and TOP005, directed against human CCR3 (Figure 1). Phosphorothioate oligodeoxynucleotides (ODNs) were designed to impart resistance to destructive cell nucleases, thereby maintaining structural integrity inside cells while inhibiting gene expression through low-stability formation duplexes with complementary RNA. TPI ASM8 was tested in the human allergen challenge model of asthma initially with a four-day treatment of 1500 mcg once daily inhaled via nebulizer. The allergen-induced levels of βc mRNA and CCR3 mRNA in sputum-derived cells were inhibited by TPI ASM8, demonstrating pharmacological and on-target effects. Compared with placebo, TPI ASM8 significantly reduced the early asthmatic bronchoconstrictor response, with a similar trend in the late asthmatic response. TPI ASM8 inhibited the allergen-induced sputum eosinophil influx by 46% and inhibited the increase in total cells by 63% after the allergen challenge [100]. A follow-up dose-response study with a four-day treatment of 1, 2, and 4 mg twice daily and 8 mg once daily, using the same allergen challenge model, demonstrated significant attenuation of all allergen-induced outcomes, early and late asthmatic responses, sputum eosinophils, airway eosinophil cationic protein (ECP) level, and methacholine PC_20_. [101] Studies on anti-CCR3 monoclonal antibodies in mouse models have also shown promising effects of this biologic on inhibiting eosinophilic inflammation in eosinophil-mediated diseases. Administering anti-CCR3 antibody in mouse model of eosinophilic gastroenteritis significantly decreased GI eosinophilic inflammation and manifestations of the disease [102]. Similar results were also seen in the BAL and lung tissue of asthmatic mouse models treated with anti-CCR3 [103].

### 4.3. CRTH2 Antagonism

Chemoattractant receptor-homologous molecule (CRTH2) is a G-protein-coupled receptor selectively expressed by type 2 T lymphocytes, basophils, eosinophils, and ILC2s [104] (Figure 1). Prostaglandin D2 (PGD2) is secreted by activated mast cells and binds to the CRTH2 receptor, resulting in the release of IL-4, IL-5, and IL-13 from Th2 cells ILC2s [105]. Preclinical studies appeared promising, with a highly selective and potent CRTH2 antagonist reducing airway hyperresponsiveness; IL-4, IL-5, and IL-13 release; airway mucus production; and leukocyte infiltration [106]. This led to an interest in developing CRTH2 antagonists as a new asthma therapy. While CRTH2 antagonists are overall safe and well-tolerated in humans, results to date have shown mixed results. The oral CRTH2 antagonist OC000459 administered twice daily was tested in a placebo-controlled double-blind, parallel group study of steroid-free asthmatics with persistent symptoms [107]. The study trial results, excluding noncompliant subjects, did show a significant improvement in FEV1 of 9.2% for subjects on OC000459 versus 1.8% on placebo (*p* = 0.037) and a reduction in mean sputum eosinophil counts from 2.1% to 0.7% (*p* = 0.03) after OC000459 treatment. Similarly, the oral CRTH2 antagonist ARRY-502 in mild atopic asthmatics found a small but significant FEV1 improvement compared to placebo (3.9%, *p* = 0.02), with improvements in ACQ-7, β-agonist use, and symptom-free days compared to placebo (*p* < 0.001, *p* < 0.001, and *p* = 0.07 respectively) [108]. Fevipiprant is an oral CRTH2 antagonist that showed promise in a single-center Phase 2 study of 61 moderate-to-severe asthmatics with elevated sputum eosinophil counts at baseline [109]. This trial showed a significant reduction in sputum eosinophil levels after fevipiprant treatment (between-group difference 3·5-fold, *p* ≤ 0.01), significant reductions in ACQ-7 score in the subgroup with poor control (*p* = 0.046, change in mean ACQ-7 = −0.37), and significant improvements in both postbronchodilator FEV1 and Asthma Quality of Life Score (AQLQ(S)) (*p* = 0.021, increased by 0.06 L and *p* ≤ 0.01 increased by 0.27 points, respectively). Building on this trial, LUSTER-1 and LUSTER-2, two replicate Phase 3 randomized, double-blind, placebo-controlled, parallel-group trials of fevipiprant were completed, primarily aimed at reducing asthma exacerbations [110]. Both LUSTER trials were completed in subjects aged 12 years or over with uncontrolled asthma despite dual or triple asthma therapy, and subjects were randomized to once-daily fevipiprant 150 mg, fevipiprant 450 mg, or placebo on a 1:1:1, with two-thirds of patients having blood eosinophil counts of 250 cells/μL or higher. The primary efficacy endpoint of annualized rate of moderate-to-severe asthma exacerbations was not met in either trial, in either dose. Pooled analysis of both studies showed a 14% reduction in annual exacerbation rate in the high eosinophil population, with a 10% reduction in the overall population for the 150 mg dose, and a 23% and 22% reduction, respectively, for the 450 mg dose. Therefore, while the results are disappointing in the T2-high patient group, a 22% overall reduction in exacerbation rate at the higher dose suggests fevipiprant could be beneficial in the T2-low group.

### 4.4. Regulation of Eosinophil Apoptosis

Sialic-acid-binding immunoglobulin-like lectin (Siglec)-8 is a cell-surface inhibitory receptor expressed selectively on human eosinophils (Figure 1) and mast cells, and it is under investigation as a therapeutic target for the treatment of allergic and inflammatory diseases [111,112,113]. The binding of a monoclonal antibody to Siglec-8 (lirentelimab/AK002) has been shown to induce death of cytokine-primed eosinophils via antibody-dependent cellular cytotoxicity (ADCC) [112]. Experiments in airway cells from asthmatic patients have demonstrated that gene expression for Siglec-8 increases in asthma, correlates with gene expression for eosinophils and mast cells, and is inversely correlated with measures of airflow obstruction. Furthermore, when airway cells were exposed to AK002 ex vivo, there was a reduction in the eosinophil population [114]. In clinical trials, the inhibitory activity mediated by AK002 has led to improvements in allergic diseases, including chronic spontaneous urticaria and eosinophilic gastritis [115,116]. For the treatment of asthma, targeting Siglec-8 appears to be a reasonable strategy to decrease sputum eosinophils, with the additional benefit of inhibiting lung mast cells. Ongoing trials are underway in patients with eosinophilic esophagitis (NCT04322708), but not in asthma at this time. Phase 2 clinical trials of AK200 (Lirentelimab) in patients with eosinophilic gastritis and eosinophilic duodenitis has presented anti-Siglec-8 antibody as a potential treatment for these patients [116]. Reports of this trial showed that the mean percentage change in gastrointestinal eosinophil count was 0.86%, and the mean change of total symptom score was −0.48% (*p* < 0.001).

## 5. Targeting Upstream Pathways of T2 Cytokines

### 5.1. GATA-3 DNAzyme

Blocking pathways leading to the production of type 2 cytokines is another promising approach. The transcription factor GATA-3 promotes the development of naïve T cells into Th2 cells, and directly induces the production of Th2 cytokines by transactivation of the promoters for IL-5 and IL-13. GATA-3 also regulates other cell types involved in bronchial asthma, including mast cells, eosinophils, basophils, and epithelial cells [117,118,119]. The central role of GATA-3 in the underlying immune pathways for the development of inflammatory allergic responses, and its significantly increased expression in the airways of asthma patients [120,121,122,123], support GATA-3 as a novel target for therapeutic intervention in type-2-driven asthma (Figure 1). DNAzymes of 10-23 RNA-cleaving family are single-stranded catalytic DNA molecules containing two substrate-recognition domains that combine the specificity of DNA base pairing and a central catalytic domain that cleaves specific sequences in a target mRNA molecule [124]. It has been reported that GATA-3-specific DNAzymes such as gd21 and hgd40 can significantly reduce GATA-3 mRNA expression and experimental asthma in vitro and in vivo [117,118].

Furthermore, GATA-3 DNAzymes are reported to not have off-target effects, especially with regard to nonspecific activation of innate immune mechanisms such as those via TLR9, activation of the NFκB pathway, or release of proinflammatory cytokines [124]. Based on the overall positive results in animal models, the GATA-3-specific DNAzyme candidate hgd40 (also called SB010 for inhaled formulation) has been developed as a novel therapeutic approach for the treatment of allergic asthma [125,126,127], and has shown excellent safety and tolerability properties in preclinical and clinical phase I studies [118,126]. In a successful phase IIa study in the allergen challenge model in mild allergic asthmatics, SB010 significantly attenuated early and late asthmatic bronchoconstriction in association with a decrease in Th2-dependent biomarkers including sputum eosinophilia, sputum tryptase, and plasma IL-5 levels [128]. GATA-3 DNAzyme is being explored in other airway diseases such as chronic obstructive pulmonary disease (COPD), where inhalation of 10 mg SB010 bid for 28 days in COPD patients significantly reduced sputum eosinophilia with a trend to lower IL-5 levels [129]. Collectively, these studies suggest GATA-3 DNAzymes could be an important new approach for the treatment of a variety of chronic inflammatory diseases.

### 5.2. Anti-TSLP

TSLP is produced by the epithelium following exposure to external stimuli such as viruses, bacteria, and allergens, and drives allergic inflammation through binding to the TSLP receptor (TSLPR) on numerous immune cells, including mast cells [130], dendritic cells [131], and eosinophils [132]. TSLP is present in increased levels within the bronchial mucosa of asthmatics compared to healthy controls [133], with TSLP expression increased within a subset of severe asthmatics despite high-dose corticosteroid therapies [134]. TSLPR deficiency in mice results in reduced ILC2 expression of IL-5, IL-13, and airway eosinophils. TSLP and IL-33 appear to work synergistically and enhance the expression of each other’s receptors on ILC2s, resulting in increased allergic inflammation [135]. Blocking TSLP in the allergen challenge model in mild allergic asthma inhibited allergen-induced early and late asthmatic responses and eosinophilic inflammation [97,136]. These data suggest that the TSLP blockade inhibits the release of proinflammatory cytokines by immune cells, and may also help prevent asthma exacerbations and improve asthma control. Due to its activity early in the inflammation cascade, blockade of TSLP may be suitable for a broad population of patients with severe, uncontrolled asthma. Moreover, a recent study done by Kabata et al. has shown that high expression of TSLP is associated with corticosteroid resistance in patients with severe asthma. This study suggests that blockade of TSLP can improve corticosteroid resistance in severe asthmatics [137].

Tezepelumab is a first-in-class, fully human anti-TSLP monoclonal immunoglobulin G2λ that specifically binds to human TSLP and prevents interaction with its receptor [138]. The US Food and Drug Administration (FDA) has recently granted Breakthrough Therapy Designation for tezepelumab in patients with severe asthma, without an eosinophilic phenotype, who are receiving inhaled corticosteroids/long-acting beta2-agonists with or without oral corticosteroids and additional asthma controllers. The Breakthrough Therapy Designation is based on the tezepelumab Phase IIb PATHWAY data [139]. The trial showed annual asthma exacerbation rate reductions of 62%, 71%, and 66% in the tezepelumab arms receiving either 70 mg or 210 mg every four weeks or 280 mg every two weeks compared to placebo (*p* < 0.001 for all comparisons), respectively. These results were observed independent of baseline blood eosinophil count or other T2 inflammatory biomarkers. This trial also showed a significant reduction in the annual asthma exacerbation rate compared with placebo in a broad population of severe asthma patients irrespective of patient phenotype, including T2 biomarker status. Building on the PATHWAY Phase IIb trial, the PATHFINDER Phase III program was initiated in the fourth quarter of 2017 with two pivotal trials: NAVIGATOR [140] and SOURCE (unpublished). The NAVIGATOR was a Phase 3, multicenter, randomized, double-blind placebo-controlled trial of patients aged 12–80 years with severe uncontrolled asthma, randomized 1:1 to receive tezepelumab 210 mg subcutaneously or placebo every 4 weeks for 52 weeks. The primary endpoint of annualized asthma exacerbation rate overall in the population showed a 56% reduction compared to placebo in the tezepelumab group (*p* ≤ 0.01), and by 70%, 41%, and 39% in patients with baseline blood eosinophil counts of ≥300, <300, and <150 cells/µL, respectively. Tezepelumab also significantly improved FEV1, Asthma Control Questionnaire-6 (ACQ-6) scores compared to placebo (*p* ≤ 0.01), with similar safety findings between tezepelumab and placebo. The tezepelumab program includes additional planned mechanistic and long-term safety trials, and appears to be a promising treatment for a broad asthma population. An inhaled TSLP antibody fragment, CSJ117, has also shown efficacy, and will be tested in a larger phase 2 clinical trial in patients with severe uncontrolled asthma (NCT04410523).

### 5.3. Anti-IL-33

IL-33 is an alarmin cytokine released by the airway epithelium following exposure to external stimuli such as viruses, bacteria, and allergens. IL-33 induces Th2 differentiation and the release of cytokines including IL-4, IL-5, and IL-13, and the activation of ILC2s, which rapidly release large quantities of IL-5 and IL-13 [141]. These cytokines promote the activation and survival of mast cells, eosinophils, basophils, and mediate innate type 2 immunity and allergic inflammation in the lungs. IL-33 levels in asthmatics compared to controls have been significantly higher within the peripheral blood, and IL-33 levels were negatively correlated to FEV1 and positively correlated to asthma severity [142]. This had led to an interest in anti-IL-33 biological agents as possible future therapies.

Early animal studies targeting IL-33 were encouraging, with a reduction in bronchoalveolar fluid eosinophilia and airway hyperresponsiveness to methacholine [143]. Phase 2 clinical studies have been disappointing to date. SAR440340 is a human IgG4P monoclonal antibody against IL-33 that was tested in a four-arm, randomized, double-blind placebo-controlled trial, a 12-week proof-of-concept trial of SAR440340 monotherapy, dupilumab, and in combination with dupilumab, in uncontrolled moderate-to-severe asthmatics despite ICS/LABA therapy [144]. SAR440340 therapy was well tolerated, and treatment significantly reduced the proportion of loss of asthma control (LOAC) events compared to placebo, as well as improved FEV1 at 12 weeks. However, dupilumab alone had a greater improvement effect on both LOAC events and FEV1 improvement, with no incremental improvement with combination therapy. Therefore it is unlikely that SARS440340 will move into phase 3 clinical studies in asthma. Similarly, a phase 2, randomized, double-blind placebo-controlled trial of GSK3772847, an IL-33 receptor antagonist, was negative. The trial showed no statistical improvement in LOAC, with 67% of patients in the GSK3772847 arm suffering LOAC, compared to 81% in the placebo arm, and more subjects within the GSK3772847 group experienced a clinically significant asthma exacerbation (13% in the GSK3772847 group vs. 7% in the placebo group (NCT03207243)). Etokimab is another humanized IL-33 antibody that has completed a Phase 2a clinical trial, in 25 adult patients with severe eosinophilic asthma despite ICS/LABA therapy (blood eosinophils ≥300/mL), randomized to receive either 300 mg of etokimab or placebo [145]. The results showed improved FEV1 (11% maximum reduction in FEV1 over placebo at day 64) and reduced blood eosinophil level (46% maximum reduction in FEV1 over placebo at day 64); no further studies are planned. While anti-IL-33 antibodies have now largely been abandoned in asthma, trials are still ongoing in other allergic diseases, including peanut allergy and atopic dermatitis.

### 5.4. Targeting ILC2s

Group 2 innate lymphoid cells (ILC2s) are part of a family of cells that lack antigen-specific receptors and link the innate and adaptive immune responses in the pathogenesis of the allergic disease [146,147]. ILC2s are found within the airways, intestines, skin, and blood [148,149], with increased ILC2 numbers in the blood of patients with allergic diseases such as asthma [150], allergic rhinitis [151], and atopic dermatitis [152]. Accumulations of ILC2s have been reported at sites of eosinophilic inflammation: in acute lesional skin in atopic dermatitis; in the upper airways within diseased mucosa and polys in chronic rhinosinusitis [153,154]; in eosinophilic pleural effusions of primary spontaneous pneumothoraxes [155]; and most notably, in the lower airways, greater numbers of ILC2s are detected in the sputum of patients with severe asthma compared to mild asthma despite high-dose oral corticosteroid therapy [156]. Human ILC2 are typically defined as lineage^−^IL-7Rα^+^NKp44^−^CD25^+^CD161^+^CRTH2^+^ [148].

ILC2s are a significant source of T2 cytokines that drive allergic inflammation. In response to aeroallergens exposure [157], parasitic [158] or viral infection [159], the bronchial epithelium releases the cytokines IL-25 and IL-33, and thymic stromal lymphopoietin (TSLP), which results in ILC2 activation [146,160]. Once activated, ILC2 cells rapidly proliferate and produce Th2 cytokines IL-4, IL-5, IL-6, IL-9, and IL-13 in abundant amounts [161] in the absence of CD4^+^ T cells [141]. IL-4 secretion is needed for Th2 differentiation, B-cell proliferation, and mast-cell activation [162]. Human studies have found increases in activated ILC2s in asthmatics’ airways 7 to 24 h after allergen inhalation challenge, with CD4^+^ T cells increased in sputum at 24 to 48 h after the allergen challenge [157]. ILC2 activation and resultant Th2 cytokine release is now considered a key event in type 2 inflammatory diseases, with the production and release of IL-5 resulting in eosinophilia, IL-13 resulting in airway mucus production and remodeling, and IL-9 promoting goblet cell hyperplasia and mastocytosis [141,163,164,165]. This demonstrates that ILC2s are critical in developing allergic diseases. Targeting and depleting ILC2s could have a significant therapeutic value, as targeting upstream T2 inflammation may provide additional treatment options for noneosinophilic asthma patients.

## 6. Conclusions

Over the past decade, anti-IL-5 therapies have been carefully evaluated and are now widely accepted for the treatment of eosinophilic asthma. While anti-IL-5 treatment has been widely approved for treating eosinophilic asthma, attenuation of broad-range inflammatory and physiological changes after allergen challenge suggests that blocking CCR3, IL-3, and GM-CSF are also important targets for the management of allergic asthma. Recent research into the underlying pathophysiology of asthma, the contribution of other inflammatory cells, and an improved understanding of upstream mechanisms have led to development of other novel therapies. These asthma therapies have been directed against interleukin 4/interleukin 13, thymic stromal lymphopoietin, CRTH2 antagonists, and the IL-3/5/GM-CSF axis, and bring the possibility of improved asthma control for patients with severe asthma.

## Figures and Tables

**Table 1:** Eosinophil-targeted therapies.

Mechanism of Action	Name	Dosing and Route	Adverse Events	Asthma Approval	Current Clinical Trial Phase	Findings	Other Populations Investigated	Ongoing Clinical Trials
**Anti-IL-5:** Binds to IL-5, preventing IL-5 from binding to the receptor on eosinophils	GSK3511294	Long-acting SC injection	Unknown	N/A	3	3 Phase 3 trials currently recruiting	Mild asthma—results pending	NCT04719832 NCT04718103 NCT04718389
	Mepolizumab	Fixed dose—100 mg SC every 4 weeks	Rarely causes hypersensitivity reactions Risk of herpes zoster infection	Uncontrolled severe eosinophilic asthma aged ≥6 years	-	↓ Exacerbations ~50% ↓ OCS use Facilitates OCS weaning ↑ FEV1	COPD EoE	NCT04075331 NCT03656380
	Reslizumab	Weight adjusted dose—3 mg/kg IV infusion every 4 weeks	Black box warning: 0.3% of patients reported anaphylaxis	Uncontrolled severe eosinophilic asthma aged ≥18 years	-	↓ Exacerbations ~50–60% ↑ FEV1	EGPA	NCT02947945
**Anti-IL-5R:** Binds to the α subunit of the IL-5 receptor on eosinophils and basophils resulting in apoptosis	Benralizumab	Fixed dose—30 mg SC every 4 weeks for 12 weeks then every 8 weeks	Rarely causes hypersensitivity reactions	Uncontrolled severe eosinophilic asthma aged ≥12 years	-	↓ Exacerbations ~ 50–70% ↓ OCS use Facilitates OCS weaning ↑ FEV1	Atopic Dermatitis EoE	NCT03563066 NCT04543409
**Anti-IL-13:** Binds to IL-13 cytokine at the binding site of the IL-13Rα receptors, preventing binding to IL-13Rα1 and α2. Lebrikizumab also blocks binding to IL-4R α.	Anrukinzumab	IV infusion every 2 weeks	No safety concerns in Phase 2 studies	N/A	-	N/A	Mild AAs—↓allergen induced FEV1 at Day 14 but not Day 35. Ulcerative Colitis	NCT01284062
	Lebrikizumab	Fixed dose—250 mg SC every 4 weeks	No safety concerns in a Phase 3 study	N/A	-	Inconsistent effect on AER across 2 phase 3 clinical trials ↑ FEV1 ↓ Feno	Atopic Dermatitis	NCT04250350 NCT04392154
	RPC4046	SC injection	Unknown in asthma population	N/A	1	N/A	EoE	NCT02098473 NCT04753697
	Tralokinumab	Fixed dose—300 mg SC every 2 weeks	Increased risk of hyper-eosinophilia	N/A	3	Inconsistent effect on annualised AER across 2 phase 3 clinical trials	Atopic Dermatitis	NCT04556461
**Anti-IL4/IL-13:** Blocks the IL-4Rα where it binds to IL-4 and IL-13, blocking IL-4 and IL-13 signalling	Dupilumab	Age and weight based—200 or 300 mg SC every 2 weeks	Rarely causes hypersensitivity reactions Increased risk of injection site reactions	Uncontrolled severe eosinophilic asthma aged ≥12 years	-	↓ Exacerbations ~50–60% ↓ OCS use Facilitates OCS weaning ↑ FEV1	Peanut Allergy CRSsNP EoE Aspirin Intolerance	NCT03793608 NCT04362501 NCT03633617 NCT04442256
	Pascolizumab	Monthly IV infusion	Unknown	N/A	-	A Phase 2 pilot study in symptomatic steroid naïve asthma failed to show efficacy.	Further development terminated	NCT00024544
	Pitrakinra	Inhalation or SC	No safety concerns in a Phase 2 study	N/A	-	A Phase 2b study found no significant difference in AER over placebo at any dose.	Further development terminated	NCT00801853
**Anti-IL-33:** Monoclonal IgG MAb that potently and specifically bind IL-33	MEDI3506	SC or IV	Unknown	N/A	2	Trial currently recruiting	COPD Atopic Dermatitis Diabetic Kidney Disease	NCT04570657 NCT04631016 NCT04212169 NCT04170543
	REGN3500	SC every 2 weeks	No safety concerns in a Phase 2 study	N/A	2	↓ LOAC compared to placebo, however dupilumab had a greater effect	COPD	NCT04701983 NCT04751487
**Anti-ST2:** MAb binds to ST2, the subunit of IL-33 receptor	Astegolimab	SC every 4 weeks	Unknown	N/A	2b	Results awaited	Atopic Dermatitis COPD Covid-19	NCT03747575 NCT03615040 NCT04386616 NCT02918019
**Anti-TSLP:** Monoclonal antibody binds to TSLP preventing its interaction with its receptor	CSJ117	Inhaled TSLP antibody fragment	Unknown	N/A	2	Trial currently recruiting	Mild AAs—↓ allergen induced responses	NCT04410523
	Tezepelumab	Fixed dose—210 mg every 4 weeks	Similar safety finding between tezepelumab and placebo *	N/A	3	↓ Exacerbations * ↑ FEV1 *	COPD Severe Steroid Dependent Asthma	NCT04039113 NCT03406078
**CRTh2:** DP2 Antagonist	AZD1981	Once or twice daily tablet	No safety concerns in Phase 2 studies	N/A	-	No change in FEV1 or asthma control	COPD	NCT00690482
	BI674800	Inhaled twice daily	No safety concerns in Phase 2 studies	N/A	-	Inconsistent effect on FEV1 and ACQ across 2 phase 2 clinical trials	Further development terminated	NCT01090024 NCT01092143
	Fevipiprant	Once daily tablet	No safety concerns in Phase 3 studies	N/A	3	AER—22% reduction in overall asthma population, 23% in eosinophil-high population	COPD—terminated	NCT03810183
	GB001	Once daily tablet	Unknown	N/A	2b	Asthma worsening or AER—no benefit	CRSsNP CRSwNP	NCT03683576 NCT03956862
	Timapiprant	Once daily tablet	No safety concerns in Phase 2 studies		2	No significant difference in sputum eosinophils or FEV1	Moderate asthma—↓ sputum eosinophils, ↑ FEV1 Atopic dermatitis	NCT02660489
**GATA-3 DNAzyme:** Specifically, and selectively targets GATA3	SB010	Inhalation once daily	No safety concerns in a small Phase 2a study	N/A	2a	N/A	Mild AAs—↓allergen induced responses COPD	
**β_c_ receptor:** blocking binding/synthesis of common β_c_ receptor –of IL-3, GM-CSGF and IL-5	CSL311	Dose ascending study ongoing	Unknown	N/A	1	N/A	Mild Asthma	NCT04082754
	TPI ASM8	Once daily inhalation	No safety concerns in a small Phase 2 studies	N/A	2b	Attenuation of allergen-induced late asthmatic response	Further development terminated	NCT01158898 NCT00550797 NCT00822861 NCT00402948
**Anti-Siglec 8:** MAb binds to Siglec-8 inducing apoptosis of eosinophils	Lirentelimab	Monthly IV infusion	Unknown	N/A	-	-	EoE Chronic Urticaria	NCT04620811 NCT04322708
Decreases eosinophil maturation	Dexpramipex-ole	Once daily tablet	Increased risk of neutropenia	N/A	2	Trial currently recruiting	CRSwNP Amyotrophic lateral sclerosis	NCT04046939
JAK inhibitor	AZD0449	Inhaled therapy	Unknown	N/A	1	N/A	Mild allergic asthma	NCT03766399
Anti-CD4 MAb that induces Treg activation	Tregalizumab	SC injection	Unknown	N/A	2	N/A	Mild allergic asthma Rheumatoid arthritis	NCT04673591

Definition of abbreviations: AA = atopic asthmatics; ACQ = asthma control questionnaire; AD = atopic dermatitis; AER = annual exacerbations rate; COPD = chronic obstructive pulmonary disease; CRSsNP = chronic rhinosinusitis without nasal polyps; EoE = eosinophilic esophagitis; IL= interleukin; IV = intravenous; LOAC = loss of asthma control; Mab = monoclonal antibody; OCS = oral corticosteroids; SC = subcutaneous. *—Based on one phase 3 trial result to date.
